# Damage amplification during repetitive seismic waves in mechanically loaded rocks

**DOI:** 10.1038/s41598-022-26721-x

**Published:** 2023-01-23

**Authors:** Anthony Lamur, Jackie E. Kendrick, Lauren N. Schaefer, Yan Lavallée, Ben M. Kennedy

**Affiliations:** 1 Department of Earth, Ocean and Ecological Sciences, University of Liverpool, 4 Brownlow Street, Liverpool, L69 3GP UK; 2 Department for Earth and Environmental Sciences, Ludwig Maximilian University of Munich, Theresienstraße, 41/III, 80333 Munich, Germany; 3 U.S. Geological Survey, Geologic Hazards Science Center, 1711 Illinois St., Golden, CO 80401 USA; 4 School of Earth and the Environment, University of Canterbury, Private Bag 4800, Christchurch, 8140 New Zealand

**Keywords:** Natural hazards, Volcanology

## Abstract

Cycles of stress build-up and release are inherent to tectonically active planets. Such stress oscillations impart strain and damage, prompting mechanically loaded rocks and materials to fail. Here, we investigate, under uniaxial conditions, damage accumulation and weakening caused by time-dependent creep (at 60, 65, and 70% of the rocks’ expected failure stress) and repeating stress oscillations (of ± 2.5, 5.0 or 7.5% of the creep load), simulating earthquakes at a shaking frequency of ~ 1.3 Hz in volcanic rocks. The results show that stress oscillations impart more damage than constant loads, occasionally prompting sample failure. The magnitudes of the creep stresses and stress oscillations correlate with the mechanical responses of our porphyritic andesites, implicating progressive microcracking as the cause of permanent inelastic strain. Microstructural investigation reveals longer fractures and higher fracture density in the post-experimental rock. We deconvolve the inelastic strain signal caused by creep deformation to quantify the amount of damage imparted by each individual oscillation event, showing that the magnitude of strain is generally largest with the first few oscillations; in instances where pre-existing damage and/or the oscillations’ amplitude favour the coalescence of micro-cracks towards system scale failure, the strain signal recorded shows a sharp increase as the number of oscillations increases, regardless of the creep condition. We conclude that repetitive stress oscillations during earthquakes can amplify the amount of damage in otherwise mechanically loaded materials, thus accentuating their weakening, a process that may affect natural or engineered structures. We specifically discuss volcanic scenarios without wholesale failure, where stress oscillations may generate damage, which could, for example, alter pore fluid pathways, modify stress distribution and affect future vulnerability to rupture and associated hazards.

## Introduction

Processes occurring in the geosphere (e.g., tectonic earthquakes^1^, magma movement^2^), hydrosphere (e.g., tides^3^, storm surges^4^), atmosphere (e.g., wind^5^), cryosphere (e.g., glacier dynamics^6^) and biosphere (e.g., anthropogenic activities: explosions, hydraulic fracturing, traffic^7^) can generate mechanical oscillations. The oscillations result from the dissipation of accumulated stresses and are propagated as elastic waves^1^. These waves vary in amplitude, frequency and duration, and travel through planets as body or surface waves. Their interactions with natural and human-made materials can result in damage and structural failure, which can generate hazards, economic losses and, in the most devastating cases, loss of life. The most common example of material failure associated with mechanical oscillations is related to seismic activity, or earthquakes. During these events, the amplitude of elastic waves is at its peak where the stress drop occurs, and with increasing distance attenuation and dispersion affect the wave amplitude/frequency^8–11^, thus leading to a spectrum of mechanical responses in the materials through which they travel.

Experimental rock physics has provided broad constraints on the mechanical behaviour of rocks subjected to different loading conditions^12,13^. In the brittle regime, material deformation proceeds differently depending on the magnitude and rate of stress applied, as well as the ambient pressure and temperature conditions^14,15^. During stressing events, laboratory experiments have shown that rocks initially experience a brief period in which pre-existing (micro-)cracks aligned sub-perpendicular to the principal stress direction, shut. Materials then deform elastically, until, as stress builds up beyond a critical yield point (termed Cʹ)^16^, cracks nucleate and propagate (in a direction near parallel to the principal applied stress) causing dilatant, inelastic deformation. Ultimately, further stressing may cause the coalescence of microfractures leading to system-scale failure^17,18^. Additionally, rocks and materials can be exposed to prolonged stressing events at constant load conditions. During these tests, rocks may deform inelastically (i.e., creep) at a rate commensurate with the magnitude of the applied stress^19^. This time-dependent, inelastic deformation results from sub-critical crack growth, and cracks can eventually coalesce, potentially leading to failure^15,19^. Tests using cyclic loading can also be performed on mechanically loaded materials to characterise their fatigue limit^20^ (i.e., the maximum number of cycles with a given stress amplitude that a material can withstand). Several cyclic (fatigue) tests have been designed to understand the build-up of damage in materials^21–26^: (1) constant amplitude waveforms around a constant mean stress; (2) increasing amplitude waveforms around a constant mean stress; (3) increasing mean stress with constant amplitude waveforms; and (4) waveforms with increasing amplitude and mean stress. In all four test types the shaking frequency may be constant or accelerating/decelerating and geometry of the waveform is typically linear, sinusoidal, or square^20^. In geosciences, such tests are commonly used to characterise mechanical response to stressing^27,28^, the effect of pore fluid pressure oscillations^22,29,30^, or thermal shock cycles^31–35^. However, while fatigue test designs may incorporate increasing amplitudes, they usually fail to investigate the effects of waveforms with amplitudes both increasing and decreasing through time, such as those observed during earthquakes.

Prolonged fatigue tests show that both engineered materials and geomaterials can rupture at stresses lower than observed during monotonic strength tests, as they accumulate damage through each stress cycle^25,36–38^, thus conferring upon these materials a fatigue limit that can be exceeded after a large number of oscillations^20,21,24,34^. The magnitude of stress reached during the oscillations is critical to damage accumulation, as materials typically accumulate damage after exceeding the stress previously experienced, a phenomenon termed the Kaiser effect^23^. Importantly, the relationships between creep mechanisms and fatigue have been shown to be intricate and dependent on a large number of variables such as stressing conditions (mean stress; oscillation amplitude and frequency)^39,40^; material physical properties (crystal size, habit, shape; pore volume and characteristics; intra-granular/pore distance)^41–43^; the presence of fluids (e.g., stress corrosion, fluid drainage)^44^ and temperature^45,46^. As such, distinguishing between the effect of creep versus fatigue mechanisms remains difficult and characterising the damage accumulated by individual oscillation events has typically only been shown qualitatively^47^.

Here, we aim to unravel the susceptibility of volcanic rocks to fatigue induced by seismic activity via a systematic quantification of the mechanical and physical impact of stress oscillation events with varying amplitude (in both space and time), such as those observed in natural earthquakes when attenuation and scattering ultimately trigger a spectrum of mechanical responses. Ultimately, we want to resolve and understand the accumulation of microstructural damage in rocks in seismically active areas, where the repetition of stress oscillations induced by earthquakes may be frequent and may prompt changes in different geological settings; and how such changes may trigger further hazards by affecting stress fields particularly around volcanic edifices where numerous and repetitive earthquakes of low amplitude are often observed.

## Materials and methods

The experimental work was conducted in the Experimental Volcanology and Geothermal Research laboratory at the University of Liverpool (the equipment is now at the LMU-Munich).

### Materials

**Figure 1:**
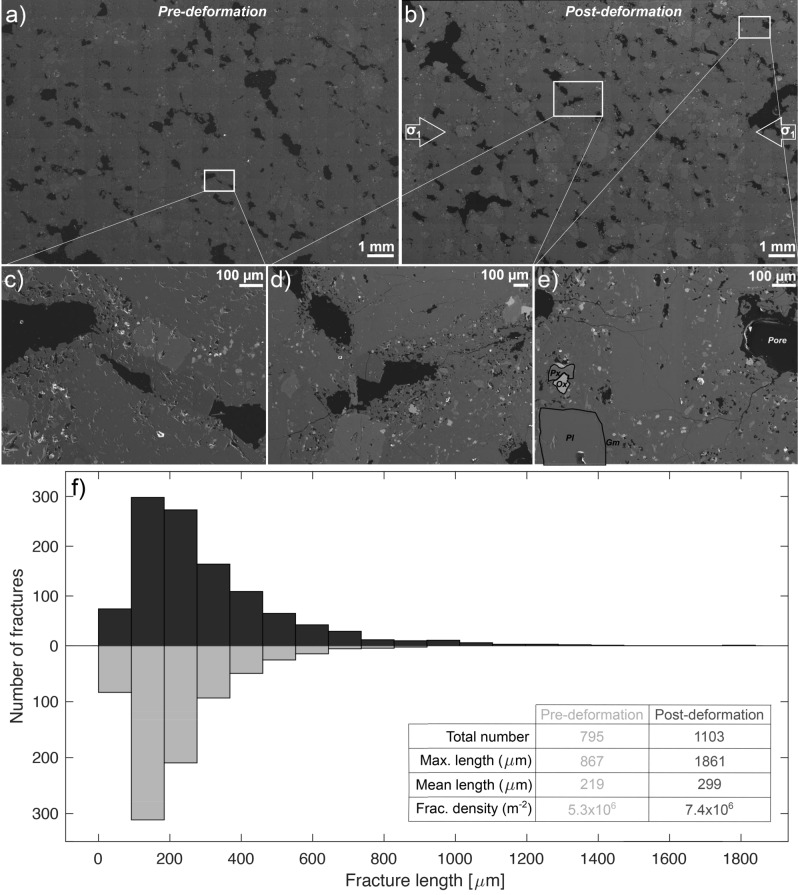
Microstructural fabrics of pre- and post-deformation materials. Backscatter electron scan of (**a**) intact material (pre-deformation) and (**b**) deformed material (post-deformation; with creep at 65% of average uniaxial compressive strength (UCS), ± 5% amplitude stress oscillation) showing phenocrysts of pyroxene (Px; light grey) and plagioclase (Pl) set in a glassy groundmass (Glm; both dark grey) with microlites of plagioclase, pyroxene and oxides (Ox; white) and pores (black). (**c**) Zoomed-in section of the intact material (from inset in (**a**)) with very low prevalence of microfractures. (**d,e**) Zoomed-in sections of the deformed material (from insets in (**b**)) showing that micro-fractures extend from the pores, sub-parallel to the applied principal stress (σ_1_, indicated in (**b**)) and traverse the groundmass and phenocrysts as a result of the experiment. (**f**) Fracture length distribution for the intact (light grey) and deformed samples (dark grey) showing that the deformed material hosts a slightly higher number of fractures and thus fracture density. Fractures in the deformed material also attain greater lengths than the pre-deformed material.

To perform a repeatable series of extended tests (i.e., creep or fatigue) we chose a well-studied, homogeneous porphyritic andesite rock, COLP2, collected from a block and ash flow deposit at Volcán de Colima, Mexico, in 2012^48^. In total, 27 cylindrical specimens 52 mm in length and 26 mm in diameter were prepared from a large block. The porphyritic andesite contains phenocrysts of plagioclase and pyroxene, with microlites of plagioclase, pyroxene and iron–titanium oxides in a glassy groundmass, consistent with lavas erupted at Colima volcano^49^. The rock hosts sub-rounded to sub-angular pores up to 3 mm in diameter, and a minor quantity of narrow fractures (see Fig. 1a,c).

### Porosity determination

Because porosity and pore structure have a first-order control on material strength^50–52^, we chose a texturally homogeneous andesite, and determined the connected porosity of each prepared specimen using an AccuPyc 1340 helium pycnometer with a 100-cm^3^ chamber^53^. The pycnometer provided a measure of the skeletal volume (V_s_) of each sample (i.e., the volume of solid rock, including isolated pore space, which is scarce in crystalline volcanic rocks) with an accuracy of ± 0.1% of the measured volume. The volume of each core sample (V_c_) was then geometrically calculated, and the connected porosity (φ) was determined via: φ = (V_c_ − V_s_)/V_c_. The 27 specimens measured had a connected porosity ranging between 11.75% and 13.22% with an average of 12.72% (see Supplementary Table 1).

### Mechanical tests

In this study, three types of mechanical experiments were undertaken using an Instron 8800 uniaxial press: (i) uniaxial compressive strength (UCS) tests, (ii) creep tests, and (iii) oscillation tests under creep loads. During the tests, the stress was monitored with an accuracy of 0.02 MPa (estimated from standard error and taking into account load cell accuracy and sample dimensions), and the axial displacement was monitored at an accuracy of 10^–5^ mm. Using the monitored displacement at a given load, the axial strain was calculated, after correcting for the machine compliance (of the piston assembly) recorded at the same loading conditions.

**Figure 2:**
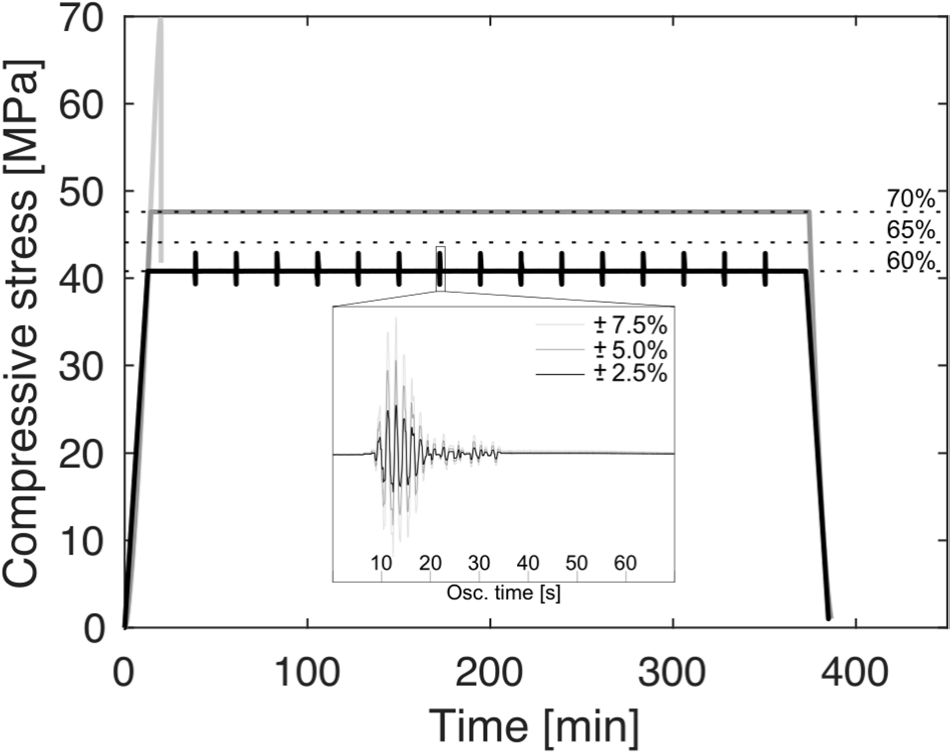
Experimental approach. The light grey curve shows the stress evolution during one of the uniaxial compressive strength (UCS) tests. The dotted lines represent the range of test set points used for proceeding experiments, at 60, 65, and 70% of the average UCS. The dark grey curve shows an example of the stress evolution during a creep experiment with a load hold of 70% of the average UCS. The black curve shows an example of the stress evolution during an oscillation test composed of 15 stressing waves applied at 22-min intervals during a load hold of 60% of the average UCS. The inset shows examples of the waveforms applied. For each oscillation test, one amplitude variation is selected, corresponding to ± 2.5% (black), ± 5% (dark grey) or ± 7.5% (light grey) of the original creep load applied.

For the UCS tests, samples were axially loaded at a constant strain rate of 10^–5^ s^−1^ until they underwent system-scale failure to define the strength of the materials and set the conditions for the subsequent tests. For the creep tests, samples were first loaded at a constant strain rate of 10^–5^ s^−1^ to the desired creep condition (i.e., to stresses corresponding to 60, 65, or 70% of the UCS value of the rock; see dotted lines in Fig. 2). The stress was then held constant for 6 h before unloading at a constant strain rate of 10^–5^ s^−1^. The oscillation tests were run at the same loading/unloading conditions as the creep tests; however, during the 6-h creep stage, 15 repeated mechanical oscillations of set amplitude were introduced every 22 min. In order to simulate the variable oscillations in strain encountered by volcanic rocks during natural earthquakes, we used a nominal waveform produced by stacking 181 seismic waves from an earthquake swarm (during which both source location and mechanism remain relatively constant) recorded between 01/10/2015 and 15/11/2015 at Unzen volcano, Japan^54^. The stacked waveform was then normalised in both time and absolute amplitude before being integrated into the Wavematrix 2.0 software of Instron. The normalised waveform was then used to control the actuator (i.e., the piston motion) by multiplying the amplitude by the desired peak load, set as a fraction of the load hold condition (2.5, 5.0, and 7.5%; see insert in Fig. 2) around a constant mean load value corresponding to a given creep condition (60, 65, or 70% of the UCS; Fig. 2). The duration of the applied load oscillation matched that of the original stacked waveform, equating to a dominant shaking frequency of ~ 1.3 Hz. We note that our experimental setup can only recreate compression/decompression around that mean load value, and thus we simulate a transverse motion in the direction of the pistons’ axis.

### Young’s modulus determination

As a proxy to assess the resultant changes in mechanical behaviour imparted by creep and mechanical oscillations, the Young’s modulus was determined during the loading and unloading phases of each experiment. Similar to the short-term, long-term average method^55^, a bespoke MatLab code was used to fit linear regressions to the stress–strain data comprised in a long window (here 1/4 of the loading/ unloading phase) rolling through the data as well as for 5 smaller windows inside the long window (i.e., 1/20 of the loading/unloading phase). For any given long window, the mismatch in slope with each small window was calculated alongside the coefficient of determination, r^2^. The long window providing the highest r^2^ and smallest average mismatch was then used to automatically estimate the Young’s modulus. The same code was used for the determination of Young’s modulus during the UCS tests.

### Acoustic emissions

To resolve the development of cracking events throughout all mechanical tests, acoustic emissions (AEs) were monitored at rate of 1 GHz using a high sensitivity R50S piezoelectric transducer connected to the curved, long edge of the sample. The AEs were fed to a 2/4/6 preamplifier set to a 20-dB gain, before reaching a PCI-2 data acquisition system developed by Physical Acoustics Corporation (PAC).

### P-wave velocity

To assess the accumulation of physical damage produced by the tests, we measured P-wave velocity (Vp) for all samples preceding and immediately following each test. Two sensors were attached to opposing ends of the cylindrical specimens. The PAC PCI-2 system emitted 100 pulses through a R50S piezoelectric sensor connected to a 2/4/6 preamplifier, set to a gain of 40 dB, and another R50S piezoelectric sensor connected to the PAC PCI-2 system received the pulses. Pulses had 1 μs duration, and were sent every 5 s to avoid interactions between pulses. Vp estimates were then made by selecting the 20 most energetic pulses and finding the time delays between the 2 sensors, which were divided by the length of the sample.

### Permeability

The permeability of all samples was measured both before and immediately after each mechanical experiment using the constant-head method in a GasPerm nitrogen permeameter, developed by Vinci Technologies. The permeability was measured at three different confining pressures (0.7, 1.4, and 2.1 MPa) by setting a constant flow rate of 1.0 or 1.5 cm^3^ min^−1^, whichever was lowest to develop a minimal pressure differential of 0.5 psi (~ 3.45 kPa) across the sample. Inertial effects were calculated and were negligible for all permeability estimations.

### SEM imaging and fracture tracing

To image and quantify the microstructure of the rocks, backscattered electron (BSE) images were taken using a Zeiss GeminiSEM 450 scanning electron microscope (SEM) from the SEM Shared Research Facility at the University of Liverpool. Images with a resolution of 150 dpi were acquired using an accelerating voltage of 10 kV, beam current of 5 nA, and a working distance of 9.9 mm. For each BSE image acquired, the fractures were manually identified and traced using ImageJ. To reduce processing time, each BSE map was cut in 9 different sections of 4.99 × 3.32 mm and tracing was performed at a zoom of 150× using the segmented line tool. Fracture width and average angle were also measured using the same images, albeit at a zoom of 1200×, by averaging the length/angle of three straight lines perpendicular to the length trace.

## Results and micro-mechanical interpretations

### Intra-experiment mechanical evolution

We first characterised the UCS on three different samples (all coming from the same block) that resulted in strengths of 62.1, 69.6, and 74.0 MPa, yielding an average strength of 68.6 ± 6.5 MPa (see Supplementary Fig. 1). The UCS values served as benchmark to resolve the effects of, and set the conditions for, the subsequent extended tests (see definition in the next paragraph). The samples had an estimated Young’s modulus of 11.02, 10.92, and 10.63 GPa, respectively, yielding an average of 10.86 ± 0.2 GPa (see Supplementary Table 1).

For both creep and oscillation tests, which are referred to as extended tests when discussed together, the rocks were subjected to three nominal stresses of 41.2, 44.6, and 48.0 MPa, (equivalent to 60, 65 and 70% of their UCS). The magnitude of the stress during oscillation was set as a fraction (± 2.5, 5.0 or 7.5%) of the aforementioned nominal target stresses (see black line and insert in Fig. 2). Here, all but one samples remained coherent after these tests; one of the two samples tested at the most extreme conditions (a creep stress of 70% of the UCS and oscillations of 7.5% of the nominal target stress) underwent rupture between the 14th and 15th oscillations.

**Figure 3:**
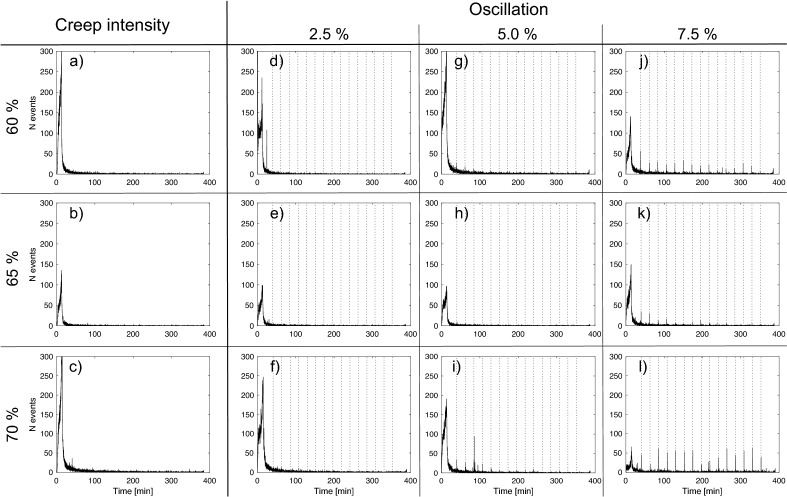
Acoustic emission profiles for the different conditions tested. (**a–c**) Show the acoustic emission count distribution through time during creep tests at 60, 65 and 70% of the average UCS, respectively. (**d–l**) Show the acoustic emission (AE) count distribution recorded during oscillation tests (also at 60, 65, and 70% of the average UCS) with amplitudes of ± 2.5, 5.0, and 7.5% stress respectively. Vertical dashed lines mark the occurrence of stress oscillations, which are accompanied by AEs at higher creep stresses and larger oscillation amplitudes.

During creep tests and oscillating stress tests, AEs were monitored as a proxy for the occurrence of micro-fracturing events^41,56–58^ (Fig. 3, Supplementary Fig. 2). In the creep tests, most AEs were generated during the loading phase, and few AEs were detected during the constant load phase (reducing from the onset of the hold; Fig. 3a–c). This indicates that during the initial loading phase, pre-existing micro-fractures shut perpendicular to the primary applied stress direction, σ_1_ and micro-fractures were created/ modified parallel to σ_1_, and that during creep very few micro-cracks nucleated and grew at a detectable scale. This is consistent with previous creep experiments that showed that only small amounts of subcritical crack growth occur before reaching tertiary creep deformation^19^. During oscillation tests we noted a similar preponderance for AEs during the loading phase (Fig. 3d–l). However, we also monitored bursts in AE activity when mechanical oscillations shook the samples (outlined by dotted lines on Fig. 3d–l); we observed that the number of AEs in these bursts increases with both the stress level of background creep and with the amplitude of the oscillations. These bursts in AEs indicate that either new fractures are nucleated and/ or existing cracks propagate and coalesce. For the sample that underwent rupture between the 14th and 15th oscillations, we observed an acceleration in AE before rupture (see Supplementary Fig. 2l). We also observed that the ± 2.5% amplitude tests did not seem to generate AE bursts. Additionally, only the first few oscillation events during the ± 5% amplitude tests registered AE bursts. In both cases, this could be due to the fact that the sensors were not able to record the smallest cracking events and thus we cannot rule out the occurrence of cracking due to these small oscillations.

**Figure 4:**
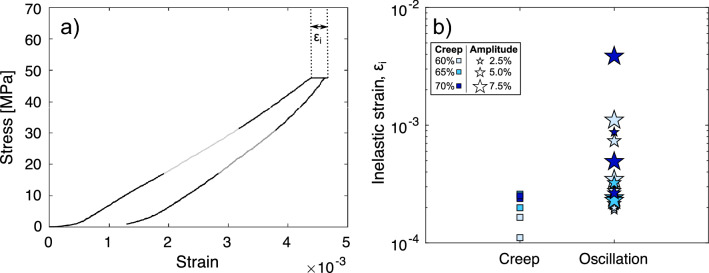
Strain response to mechanical testing. (**a**) Stress–strain curve for a creep test illustrating how the strain (ε_i_) at creep is calculated. The light and dark grey sections during the loading and unloading phase respectively show where Young’s modulus (E) was calculated. (**b**) Strain during creep (ε_i_; during the load-hold portion) for the different test types (squares are creep tests, stars are oscillation tests). For both creep and oscillation tests the different shades of blue correspond to the different target stresses of the creep portions (the darker the shade, the higher the stress). For oscillation tests, the size of the star depicts the amplitude variation of the stress oscillations with small stars corresponding to ± 2.5%, intermediate to ± 5%, and large to ± 7.5% change from the creep stress.

The creep and oscillation tests result in the observation of inelastic (permanent) strain (${\upvarepsilon}_{i}$) in the materials. The amount of inelastic strain during the 6-h creep period of both types of extended tests (creep and oscillation) is determined by the difference between the maximum accumulated strain and the strain at the start of the creep period (i.e., following the initial loading phase; Fig. 4a; see also Supplementary Figs. 3–5 for the raw mechanical data). It is worth noting that the stressing conditions for the creep and oscillation tests largely overlap, as oscillation amplitudes are a small percentage of the creep condition, and thus there are substantial overlaps within the observed mechanical results. However, careful examination of each monitored and measured parameter enables us to establish various trends, which we discuss holistically here. For creep tests, the results show that the amount of inelastic strain increases with applied creep stress (see squares in Fig. 4b; darker colours depicting higher creep loads). When mechanical oscillations were introduced, the sample underwent up to four times more inelastic strain than under creep conditions alone (at least for samples that did not lead to system-scale failure), and more than ten times after the 14th oscillation in the sample that ultimately underwent failure (see stars in Fig. 4b). However, there did not seem to be a clear correlation between the accumulated strain and the amplitude of the oscillations for a given nominal creep stress. Additionally, and contrary to the creep tests, the intensity of the applied creep stress during oscillation tests does not show a correlation with the maximum inelastic strain.

**Figure 5:**
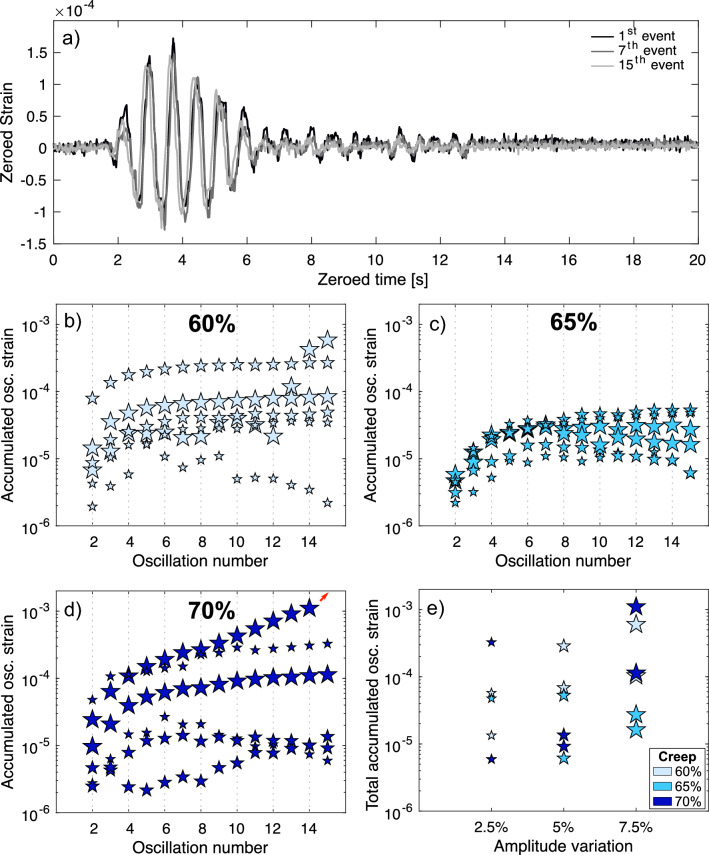
Inelastic strain accumulation caused by stress oscillations. (**a**) Strain response comparison between different stress oscillation events. Accumulated strain generated by the 15 stress oscillations during each of the tests at (**b**) 60%, (**c**) 65% and (**d**) 70% of the average uniaxial compressive strength (UCS); The red arrow indicates the trend followed by the sample that failed after 14 oscillation event. (**e**) Total accumulated inelastic strain after the final oscillation for all the creep conditions, plotted as a function of the amplitude of the stress oscillations. In (**b–e**) the different shades of blue correspond to different stress at each creep condition (the darker the shade, the higher the stress), and the size of the star depicts the amplitude of the stress oscillation (small stars correspond to ± 2.5%, intermediate to ± 5%, and large to ± 7.5%).

To investigate the development of inelastic strain imparted by individual mechanical oscillations further, we opted to filter out the creep deformation from the oscillation test samples by computing the average strain response at each creep condition (see Supplementary Information: Dynamic creep removal and Supplementary Fig. 6). We thus first cropped the strain response to each stressing event by finding the most prominent peak in the stress signal; cropping is done by selecting strain data 0.2 s before (100 data points at a sampling rate of 50 Hz) and 30 s after (1500 data points at a sampling rate of 50 Hz) the most prominent stress peak. By filtering out the creep deformation we obtained the zeroed strain response during each oscillation (Fig. 5a); we observed that the zeroed strain signal of each oscillation event became slightly compressed along both amplitude and time axes when compared to the “pristine” first straining event. Using dynamic time warping^59,60^ to match each individual oscillation event to the first reference or “pristine” event then allowed us to calculate the inelastic strain accumulated by the sample when the oscillation events occurred (i.e., the length of the shortest warping path between the two strain signals) at the different conditions tested. Examining the strain accumulated by the samples during oscillations, we observe that the first few oscillations (up to 4–6) always accumulate the most strain, regardless of the amplitude or creep stress (Fig. 5b–d). Subsequent oscillations then appear to impart a stable amount of inelastic strain, but in some cases, events progressively accumulate smaller amounts of damage than earlier oscillation events. For the sample that failed (creep stress of 70% of UCS and oscillations of ± 7.5% amplitude), the strain accumulated at each event began to increase again after the initial reduction. It is worth noting that for the sample that underwent failure we considered the last cycle to be the 14th oscillation. Similarly, for a sample tested at a creep load of 60% of UCS and subjected to oscillations of ± 7.5% amplitude (Fig. 5b), each oscillation event imparted more inelastic strain than the previous event, thus showing an acceleration in inelastic strain build up as samples approached failure under high loads, similar to that experienced during tertiary creep^19,61,62^. Although the reason for this specific sample exhibiting this response is unknown, it may be due to a higher starting micro-fracture density that would have promoted fracture coalescence. Finally, the amplitude of the oscillations seems to slightly correlate with the total amount of inelastic strain experienced by a rock (Fig. 5e), as samples subjected to the larger amplitudes tended to record higher total accumulated strains after the last straining event, especially the samples that experienced the largest, ± 7.5% amplitude, oscillations.

**Figure 6:**
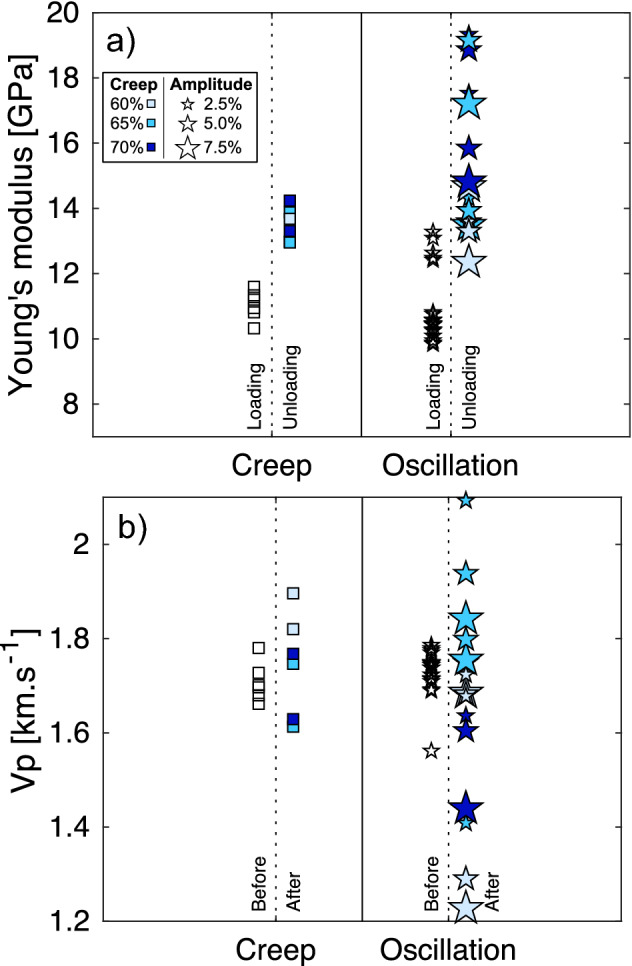
Changes in Young’s modulus and P-wave velocity (Vp) before and after the creep period. (**a**) Young’s modulus during the loading phase (hollow symbols) and unloading phase of creep (squares) and oscillation (stars) tests. (**b**) Ultrasonic velocities, Vp, estimated in pre-test samples (hollow symbols) and post-test samples deformed in creep (squares) and oscillation (stars) tests. For both creep and oscillation tests, the different shades of blue correspond to different stresses at each creep condition (the darker the shade, the higher the stress). For oscillation tests, the size of the star depicts the amplitude of the stress oscillation, with small stars corresponding to ± 2.5%, intermediate to ± 5%, and large to ± 7.5% of the creep stress set-point.

To assess the mechanical changes imparted during the different tests, the Young’s modulus was calculated during the loading (empty squares and stars) and unloading (filled squares and stars) phases of creep and oscillation tests (Fig. 6a). Loading Young’s Modulus values are consistent across UCS, creep and oscillation tests with an average value of 11 GPa and standard deviation of 0.97 GPa. These show higher values during unloading with an average of 14.85 GPa and a standard deviation of 2.09 GPa (see also Supplementary Table 1). This apparent stiffening may be explained by the closure of microfractures perpendicular to the applied stress during loading, which accommodates more strain at a given stress than the elastic deformation of the rock, and by the nucleation, growth, and coalescence of micro-cracks parallel to the applied stress. Subsequently, as the material is unloaded, asymmetric reverse sliding along these micro-cracks can alter the bulk mechanical response of the material and confer an hysteresis during cycles of loading/unloading in uniaxial conditions^63,64^. Similarly to the results presented above, we observe that oscillation tests show a more drastic change in Young’s Modulus, thus indicating that those stressing events contribute alongside creep to introduce damage in the samples.

### Induced damage impact on physico-mechanical properties

Examination of the micro-structure of the starting material and a deformed sample (65%; ± 5%) show that the experiment modified the architecture of the fracture network (Fig. 1b,d–f; see also Supplementary Figs. 7, 8). The original material contains micro-fractures that are generally restricted to either the groundmass or the phenocrysts (i.e., the fractures do not cross the boundaries; Fig. 1a,c). Additionally, the undeformed sample exhibits a fracture density of 5.3 × 10^6^ m^−2^, and the longest fracture measured reaches 867 µm (Fig. 1f). In contrast, fractures in the sample that has been subjected to oscillations tend to emanate from heterogeneities and cross both groundmass and phenocrysts alike (Fig. 1d,e). This sample exhibits a fracture density of 7.4 × 10^6^ m^−2^, and the longest fracture reaches 1861 µm (Fig. 1f). Overall, fractures cross groundmass and phenocrysts without deflection, indicating physical damage imparted by both creep and fatigue mechanisms in contrast to the pre-existing fractures commonly observed in rocks from Volcán de Colima, which are likely associated with cooling of the lava after eruption^31^.

Ultrasonic velocities of the compressional waves, Vp, were used to assess the material’s integrity after the tests. The range of Vp was slightly higher following creep tests (Fig. 6b), as some values appear to have increased (particularly at low creep stresses), whilst others decreased. The Vp of materials following the oscillation tests showed broader range than the original materials, or samples deformed under creep loads, with samples showing either an increase or a decrease in Vp. The Vp of materials following both the creep and the oscillation tests showed broader ranges than the original materials, with samples showing either an increase or a decrease in Vp. This indicates that some of the tests may have densified the rocks and increased Vp, possibly due to fracture closure, whereas other samples may have predominantly dilated, possibly due to fracture creation, reducing Vp^65^. The increase in spread of Vp following the oscillation tests was more significant than in creep tests, suggesting greater microstructural rearrangement of these samples. Vp was measured along the long axis of the samples, parallel to the principal applied stress, which is also the predominant orientation of induced fractures, therefore their impact on the measured Vp is limited. However, in combination with changes in Young’s modulus, the Vp results suggests (Fig. 6) that the passage of mechanical oscillations in a rock mass amplifies the effect of creep deformation and imparts further damage that modifies the microstructure, and importantly increases the anisotropy of the fracture network and the resultant mechanical properties of rocks.

**Figure 7:**
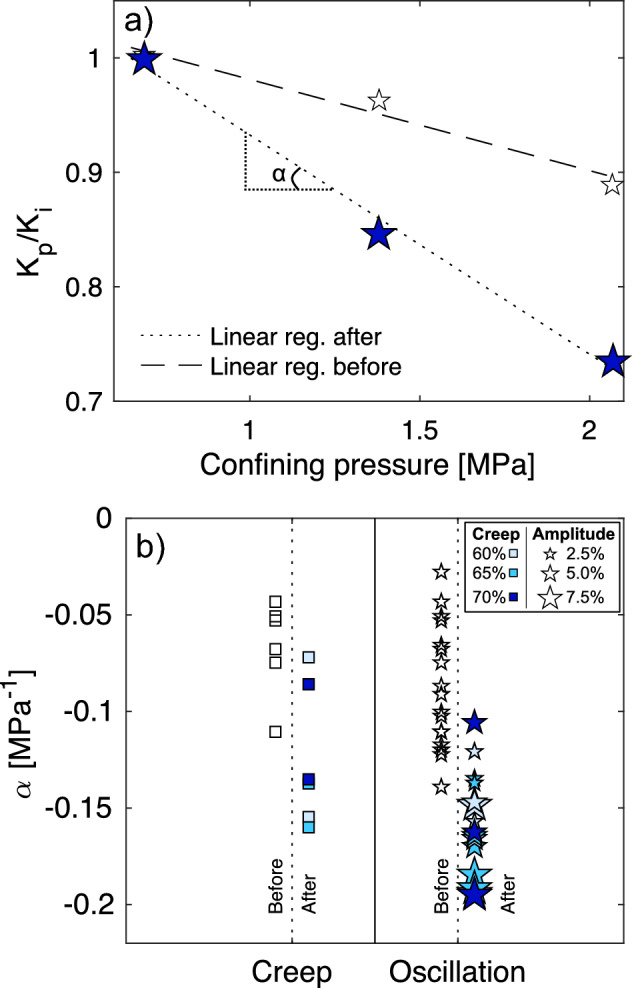
Pressure dependence of permeability in pre- and post-test samples. (**a**) Normalised permeability (K_p_ is the permeability at a given confining pressure, K_i_ is the permeability at the lowest confining pressure, 0.7 MPa) as a function of confining pressure for a sample pre-test (hollow stars) and post-test (blue stars). The dashed and dotted lines represent the linear regressions calculated for pre- and post-test samples, respectively. The permeability pressure dependence, or permeability change rate, α, corresponds to the slope of the linear regression. (**b**) Permeability change rates, α, (as explained in **a**) calculated before (hollow symbols) and after deformation for samples in creep (squares) and oscillation (stars) tests. For both creep and oscillation tests, the different shades of blue correspond to different stresses at each creep condition (the darker the shade, the higher the stress). For oscillation tests, the size of the star depicts the amplitude of the stress oscillation, with small stars corresponding to ± 2.5%, intermediate to ± 5%, and large to ± 7.5% of the creep stress set-point.

The presence of micro-fractures is an important control on permeability^66^, which, in turn, accommodates the presence and flow of pore fluids that affect the stress and, potentially, the resultant behaviour of materials^12^. As the damage created during the extended tests was generally microscopic (i.e., only samples which underwent rupture showed macroscopic damage), we did not anticipate large changes in absolute permeability values^67,68^; hence, we focused our attention on the pressure dependence of the permeability in the original materials versus experimental products, as fracture geometry (which controls permeability; e.g., aperture, connectivity, tortuosity) is pressure dependent^69^. We quantify the permeability change rate, ⍺, as a metric for relative changes in the fracture characteristics of our samples, by fitting a linear regression through the permeability values as a function of confining pressure (Fig. 7a). The permeability is normalised to the permeability at the minimum confinement of 0.7 MPa to remove the effect of sample variability and allow comparison between samples that were deformed at different conditions (see Fig. 7b). The starting materials exhibit a range of ⍺ due to material variability, with values generally above − 0.15 MPa^−1^. Following creep tests, we note a clear reduction in ⍺, indicating a higher susceptibility of permeability to reduction under confinement. Following the oscillation tests, ⍺ drops even more substantially, showing a greater increase in the dependence of permeability on confining pressure. The changes in fracture morphology (Fig. 1, Supplementary Figs. 6, 7) explain the observation of increased susceptibility of permeability to confining pressure, as the longer fractures generated parallel to σ_1_ (hence perpendicular to confinement in the permeameter) would be more effectively closed under confining pressure. Moreover, there appears to be a positive correlation between ⍺ and the oscillation amplitude. This supports the observations from the monitored AE that mechanical oscillations enhance the effect of creep and impart fracture damage that changes the physico-mechanical properties of rocks, and further indicates that the amplitude of stress oscillations effects the architecture of the permeable porous network.

### Impact of sample variability

The failure mechanism of rocks is primarily affected by microstructural arrangements (pore/crystal/micro-fracture size, orientation, geometry, and spatial relationship to one another)^17,50,62,63^. As such, every sample has a unique microstructural arrangement with an inherent variability. Therefore, sample variability may hinder direct sample to sample comparison, limiting our ability to interpret specific contrasts between individual test results. However, when our data are considered as a whole, we observe quantifiable signals and correlated trends that indicate that sample variability only has a minor effect on the results we present here, leading to overlapping ranges of values but not obscuring trends across the datasets.

## Implications

High-amplitude mechanical oscillations can trigger structural failure in both natural and man-made materials^70,71^. This effect is particularly noteworthy for materials located in the direct vicinity of large stress disturbances (e.g., close to the focus of an earthquake), and it lessens with distance, as attenuation and dispersion reduce the amplitude of the stress oscillation^1^. Our experimental campaign has shown that mechanical oscillations can systematically modify the physical and mechanical properties of rocks, and in extreme cases, prompt rupture. Here, using dynamic time warping on the recorded strain signals, we isolated the effect of fatigue mechanisms caused by individual oscillations from the effect of creep to show the build-up of inelastic strain caused by mechanical oscillations (even when the maximum stress is below the compressive strength of the material). Such mechanical pulses contribute to the generation of micro-cracks sub-parallel to σ_1_ and thus act to amplify the effect of time-dependent deformation in materials otherwise subjected to constant creep stresses (tectonic, gravitational). Importantly, although we were able to distinguish between, and quantify, the physical impact of mechanical oscillations during fatigue stressing over that of creep deformation, we could not resolve which specific mechanisms (e.g., wear along crack edges^13,64^, grain indentation^41,42^, etc.) were operating during our experimental campaign. We surmise that the amplification of physical damage by mechanical oscillations can modify the stress fields in rock masses^72^ at a wide range of temporal and spatial scales without necessarily prompting rupture or substantial deformation. For example, stress transfer along faults after an earthquake can be attributed to direct fault-fault interactions^73^ as well as to the redistribution of fluids and pore pressure^74^ that, as we show here, would be facilitated by the creation of damage due to mechanical oscillations. Our results indicate a fracture-driven anisotropy of the samples caused by deformation (Fig. 1) and accordingly, a change in the sensitivity of permeability to confining pressure (Fig. 7b). This mirrors field studies that have shown that aquifers and confining units may develop permeability anisotropy due to the accumulation of inelastic strain following an earthquake^75,76^, a process that can also affect hydrothermal and geothermal systems^77–80^. Additionally, changes in water level documented in wells after earthquakes have been attributed to changes in permeability^81,82^.

Earthquakes have long been inferred to interact with volcanoes, potentially affecting volcanic activity^83,84^ (see^80^ for a review). Whilst mechanical oscillations have been suggested to interact with magma^85,86^, here we explore how they could indirectly affect volcanic activity via damage accumulated in the wallrock. At Villarrica volcano in Chile, a moment magnitude (M_w_) 8.3 tectonic earthquake rapidly and locally prompted rupture extending to the Earth’s surface, and this has been inferred to have prompted the onset of an eruptive episode 17 days later^84^. Similar volcanic unrest was also reported in the following months at nearby Copahue and Nevados de Chillán volcanoes^84^. The trigger of the unrest at these three volcanoes was attributed to the remobilisation of fluid in the adjacent hydrothermal systems (i.e., at a scale much wider than the fault). Here, we posit that the development of permeability anisotropy caused by damage accumulation associated with mechanical oscillations during the earthquake remains a plausible explanation for the inferred redistribution of fluids in the hydrothermal system. Similarly, the M_w_9 Tōhoku earthquake in Japan is thought to have modified the stress field around Mount Fuji several hundreds of kilometres away^87^ potentially prompting failure of the wall rock around a magmatic storage zone and thus triggering magmatic injection. The accumulation of damage caused by the mechanical oscillations could potentially have enhanced the coalescence of fractures in an already highly stressed (thermal stresses, fluid migration) rock mass.

Earthquakes may also jeopardise the structural stability of prominent rock masses in non-volcanic environments, as demonstrated by observations of widespread landslides and rock-falls that may follow individual earthquake events^88–91^. It has been suggested that changes in pore fluid pressure^92–94^ and accumulation of damage^89,95^ cause this instability of rock masses, resulting in post-seismic landslides^90^. Here, we advance that the accumulation of inelastic strain and fracture damage during episodes of earthquake activity may, in some instances, amplify creep deformation, prompting sufficient changes to the materials and their pore fluids to increase their susceptibility to undergo wholesale rupture, possibly generating landslides or other types of failure-based hazards. This effect has particularly been observed during similar experiments on variably porous volcanic rocks from Unzen volcano^96^ (a volcano in Japan that historically experienced a deadly, large sector collapse event following, in the same day, the 1792 M6.4 Shimabara-Shigatusaku earthquake^97^): In this study, the authors found that depending on the initial fracture network geometry, mechanical oscillations were not only able to generate damage, but also substantially contribute to the acceleration of the deformation.

Finally, beyond natural environments, mechanical oscillations can affect the stability of anthropogenic structures, such as roads^98^, buildings^99^, bridges^100^, and dams^101^. At their most extreme, large mechanical oscillations, such as those generated by earthquakes, can lead to catastrophic collapse of engineered structures. However, the accumulation of inelastic strain caused by the repetition of low amplitude elastic waves may also severely hamper the resilience of man-made structures. For instance, road traffic can generate highly repetitive, low amplitude elastic waves^102,103^ that are able to induce inelastic strain due to damage accumulation in nearby roads, bridges, and buildings; these are important considerations in building regulations as ultimately, mechanical oscillations may weaken constructional materials, and prompt failure^104^.

Despite only using transverse piston motion, our findings indicate that testing the mechanical properties of rocks more frequently under oscillatory stress conditions simulating natural earthquakes would be beneficial in resolving their response and could increase our ability to model the storage of resources in reservoir rocks, as well as the structural stability and collapse of large rock masses, which may generate hazards, such as landslides and volcanic eruptions. In nature, we surmise that this effect may be enhanced by the passage of longitudinal waves creating additional shear in rock masses. We also caution that numerous strategies may be necessary to detect the mechanical weakening of materials caused by oscillatory stresses, and in particular note that increased anisotropy may be a particularly defining attribute of earthquake-damaged materials. Recent studies have shown that rocks with anisotropic fabrics may be particularly susceptible to mechanically induced weakening and failure^53^, which indicate that repetitive earthquakes that induce anisotropy may perpetuate non-linear, amplifying damage towards wholesale failure, as observed in this study.

## Supplementary Information

Supplementary Information.

## Data Availability

The datasets generated and/or analysed during the current study are available in the Damage Amplification repository on GitHub, https://github.com/Volcanthony/Damage-amplification.
