# Supplementary material for: Damage amplification during repetitive seismic waves in mechanically loaded rocks
Source: Sci Rep. 2023 Jan 23;13:1271. doi: 10.1038/s41598-022-26721-x (PMC9870869; doi:10.1038/s41598-022-26721-x)
Supplement: Supplementary file 1 — Supplementary Information. [file 41598_2022_26721_MOESM1_ESM.docx]

**Damage amplification during repetitive seismic waves in mechanically loaded rocks**

Anthony Lamur*^1,2^, Jackie E. Kendrick^1,2^, Lauren N. Schaefer^3^**^,^**^4^, Yan Lavallée^1,2^ and Ben M. Kennedy^4^

^1^Department of Earth, Ocean and Ecological Sciences, 4 Brownlow Street, L69 3GP, University of Liverpool, Liverpool, United Kingdom

^2^Department for Earth and Environmental Sciences, Theresienstraße, 41/III, 80333, Ludwig Maximilian University of Munich, Germany

**^3^**U.S. Geological Survey, Geologic Hazards Science Center, 1711 Illinois St., Golden, Colorado, 80401, USA

^4^School of Earth and the Environment, University of Canterbury, Private bag 4800, Christchurch, 8140 New Zealand

*anthony.lamur@min.uni-muenchen.de

**Supplementary Tables and Figures:**


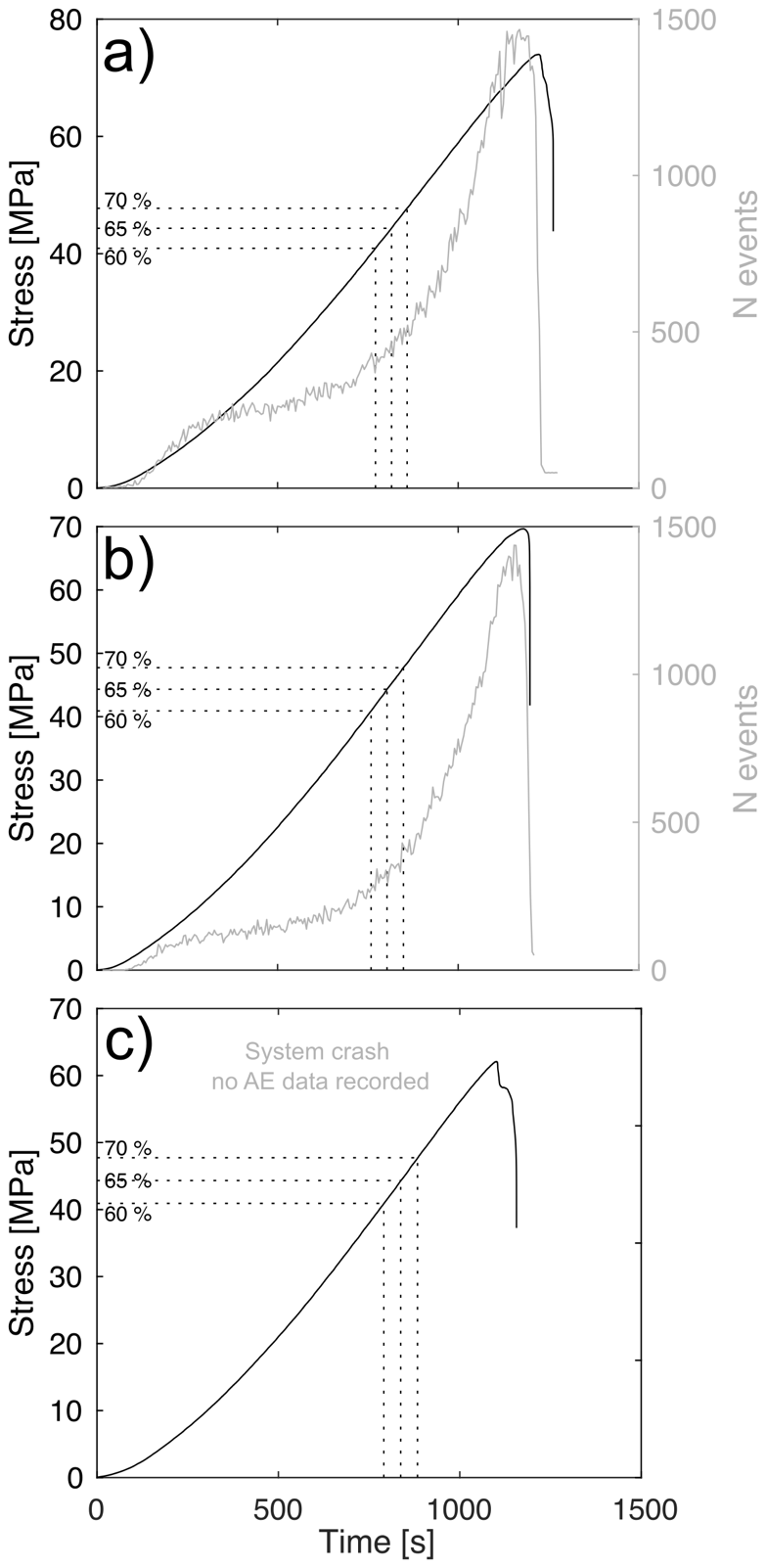


**Supplementary Figure 1. Mechanical and AE data for UCS tests**. a-c) Three Uniaxial Compressive Strength (UCS) tests, with mechanical data (black line) and cumulative AE events (grey line) through time. In all panels, the 3 dotted lines show the 60, 65 and 70% of UCS, values which were used for subsequent tests.

| **Test type** | **Stress cond. (%)** | **Amplitude (%)** | **Porosity (%)** | **Vp (m/s)** | | **Young’s modulus (GPa)** | | **Permeability (mD)** | | | | | | | |
| --- | --- | --- | --- | --- | --- | --- | --- | --- | --- | --- | --- | --- | --- | --- | --- |
|  |  |  |  |  |  |  |  | **Before** | | | | **After** | | | |
|  |  |  |  | **Before** | **After** | **Load** | **Unload** | *0.7MPa* | *1.4MPa* | *2.1MPa* | *⍺** | *0.7MPa* | *1.4MPa* | *2.1MPa* | *⍺** |
| Oscillation | 60 | 2.5 | 12.46 | 1689 | 1724 | 10.56 | 13.88 | 6.37 | 6.26 | 5.80 | -0.06 | 6.61 | 6.07 | 5.50 | -0.12 |
|  |  |  | 12.89 | 1714 | 1678 | 12.48 | 13.60 | 5.67 | 5.00 | 4.58 | -0.14 | 5.32 | 4.71 | 4.17 | -0.15 |
|  |  | 5.0 | 13.02 | 1747 | 1683 | 9.88 | 13.59 | 6.18 | 5.62 | 5.14 | -0.12 | 5.85 | 5.09 | 4.66 | -0.14 |
|  |  |  | 12.63 | 1786 | 1290 | 10.25 | 13.29 | 9.34 | 8.65 | 7.92 | -0.11 | 9.40 | 8.11 | 7.25 | -0.16 |
|  |  | 7.5 | 12.72 | 1749 | 1685 | 10.59 | 14.67 | 6.92 | 6.56 | 6.05 | -0.09 | 8.20 | 7.18 | 6.52 | -0.15 |
|  |  |  | 12.72 | 1775 | 1228 | 9.84 | 12.35 | 6.48 | 5.90 | 5.41 | -0.12 | 7.15 | 5.94 | 5.24 | -0.19 |
|  | 65 | 2.5 | 12.36 | 1779 | 2093 | 12.64 | 14.31 | 7.34 | 7.19 | 6.66 | -0.07 | 6.77 | 6.21 | 5.51 | -0.13 |
|  |  |  | 13.10 | 1723 | 1409 | 10.28 | 14.75 | 5.86 | 5.43 | 4.95 | -0.11 | 5.50 | 4.92 | 4.47 | -0.13 |
|  |  | 5.0 | 12.74 | 1738 | 1938 | 10.81 | 19.15 | 6.48 | 6.14 | 5.44 | -0.11 | 7.68 | 6.68 | 5.89 | -0.17 |
|  |  |  | 13.18 | 1738 | 1798 | 10.45 | 13.93 | 7.08 | 6.46 | 5.87 | -0.12 | 6.52 | 5.67 | 5.04 | -0.16 |
|  |  | 7.5 | 12.48 | 1770 | 1842 | 13.09 | 13.49 | 7.28 | 6.64 | 6.17 | -0.11 | 7.66 | 6.52 | 5.70 | -0.18 |
|  |  |  | 13.13 | 1750 | 1755 | 9.95 | 17.21 | 7.70 | 7.42 | 6.86 | -0.08 | 8.11 | 6.85 | 5.97 | -0.19 |
|  | 70 | 2.5 | 13.20 | 1749 | 1748 | 10.47 | 19.34 | 5.92 | 5.87 | 5.48 | -0.05 | 6.41 | 5.66 | 4.96 | -0.16 |
|  |  |  | 12.92 | 1692 | 1637 | 12.42 | 17.53 | 8.42 | 8.10 | 7.42 | -0.09 | 18.95 | 16.72 | 14.59 | -0.16 |
|  |  | 5.0 | 12.58 | 1709 | 1749 | 13.28 | 18.86 | 14.19 | 12.97 | 12.26 | -0.10 | 14.59 | 13.58 | 12.46 | -0.10 |
|  |  |  | 12.46 | 1562 | 1604 | 10.78 | 15.84 | 5.59 | 5.17 | 4.66 | -0.12 | 5.33 | 4.57 | 4.13 | -0.16 |
|  |  | 7.5 | 12.80 | 1744 | - | 10.08 | - | 7.25 | 7.11 | 6.51 | -0.07 | - | - | - | - |
|  |  |  | 13.22 | 1715 | 1439 | 10.43 | 14.82 | 5.59 | 5.31 | 4.98 | -0.08 | 6.58 | 5.57 | 4.80 | -0.19 |
| Creep | 60 | - | 12.60 | 1720 | 1820 | 10.94 | 12.98 | 7.12 | 6.70 | 6.12 | -0.10 | 6.13 | 5.91 | 5.52 | -0.07 |
|  |  |  | 11.97 | 1695 | 1896 | 11.59 | 13.68 | 5.63 | 5.16 | 4.62 | -0.13 | 4.93 | 4.37 | 3.88 | -0.15 |
|  | 65 | - | 13.11 | 1701 | 1746 | 10.32 | 13.88 | 7.80 | 7.75 | 7.34 | -0.04 | 7.96 | 6.93 | 6.21 | -0.16 |
|  |  |  | 12.82 | 1680 | 1613 | 10.81 | 12.96 | 9.05 | 8.06 | 7.24 | -0.14 | 8.00 | 7.15 | 6.49 | -0.13 |
|  | 70 | - | 12.27 | 1780 | 1768 | 11.20 | 13.31 | 6.27 | 5.98 | 5.42 | -0.10 | 7.70 | 5.13 | 4.64 | -0.28 |
|  |  |  | 12.46 | 1662 | 1629 | 11.33 | 14.23 | 7.52 | 7.49 | 6.87 | -0.06 | 6.81 | 6.60 | 6.00 | -0.09 |
| UCS | - | - | 11.75 | 1750 | - | 11.02 | - | 7.50 | 7.83 | 7.20 | -0.03 | - | - | - | - |
|  |  |  | 12.80 | 1791 | - | 10.92 | - | 6.86 | 6.20 | 5.59 | -0.13 | - | - | - | - |
|  |  |  | 13.07 | 1764 | - | 10.63 | - | 5.83 | 5.86 | 5.42 | -0.05 | - | - | - | - |

**Supplementary Table 1. Sample physical characteristics pre-/ syn- and post- deformation.**

*Calculated from permeability values normalised to the permeability measured at 0.7 MPa


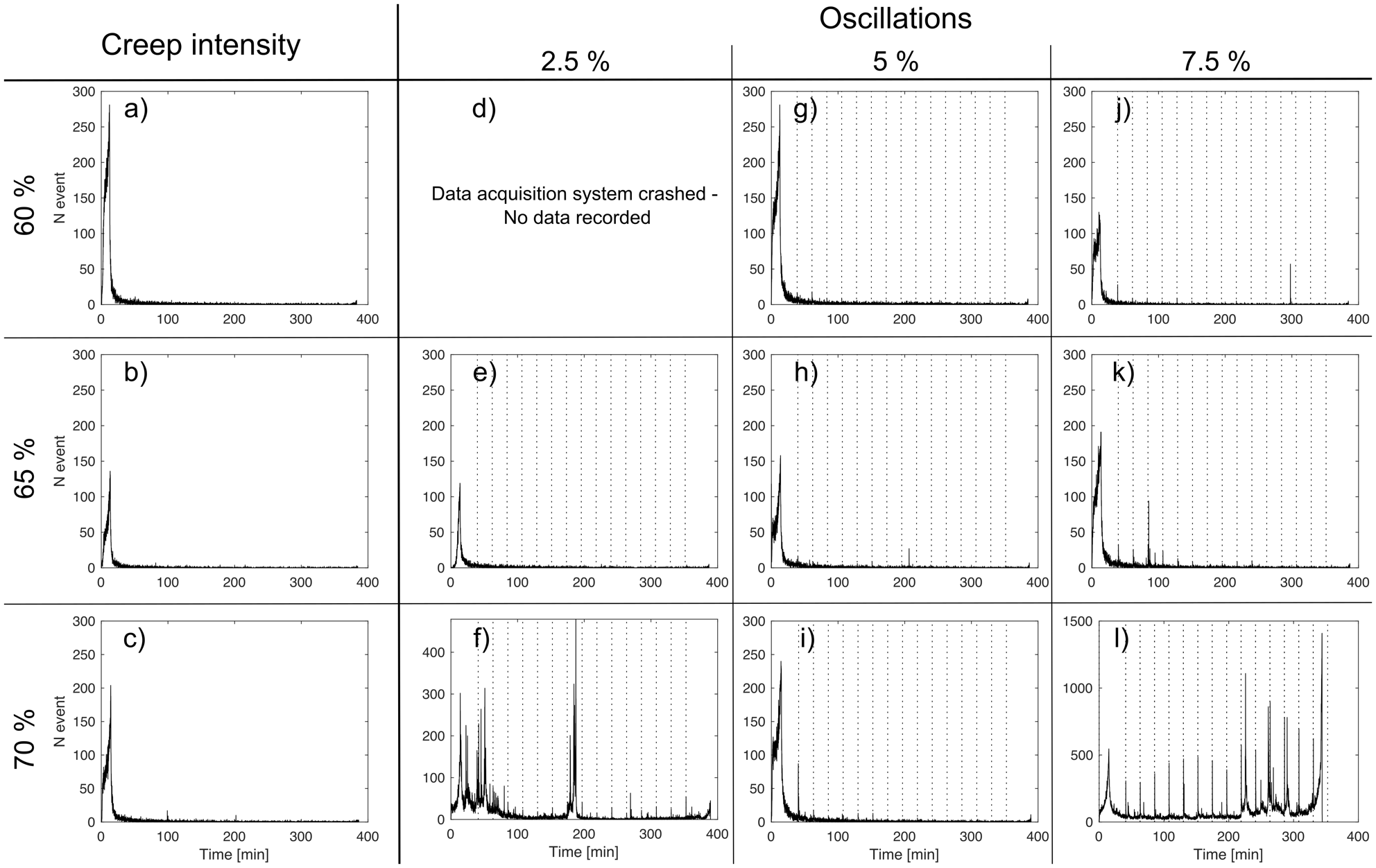


**Supplementary Figure 2.** **Acoustic emission profiles for the different conditions tested**. a), b) and c) show the acoustic hit distribution through time during creep tests at 60, 65 and 70% of the average UCS, respectively. d) – f); g) – i) and j) – l) show the hit distribution recorded during oscillation tests (also at 60, 65 and 70% of the average UCS) with amplitudes of ±2.5, 5.0 and 7.5%, respectively. The data show the repeat experiments of those shown in Figure 3.


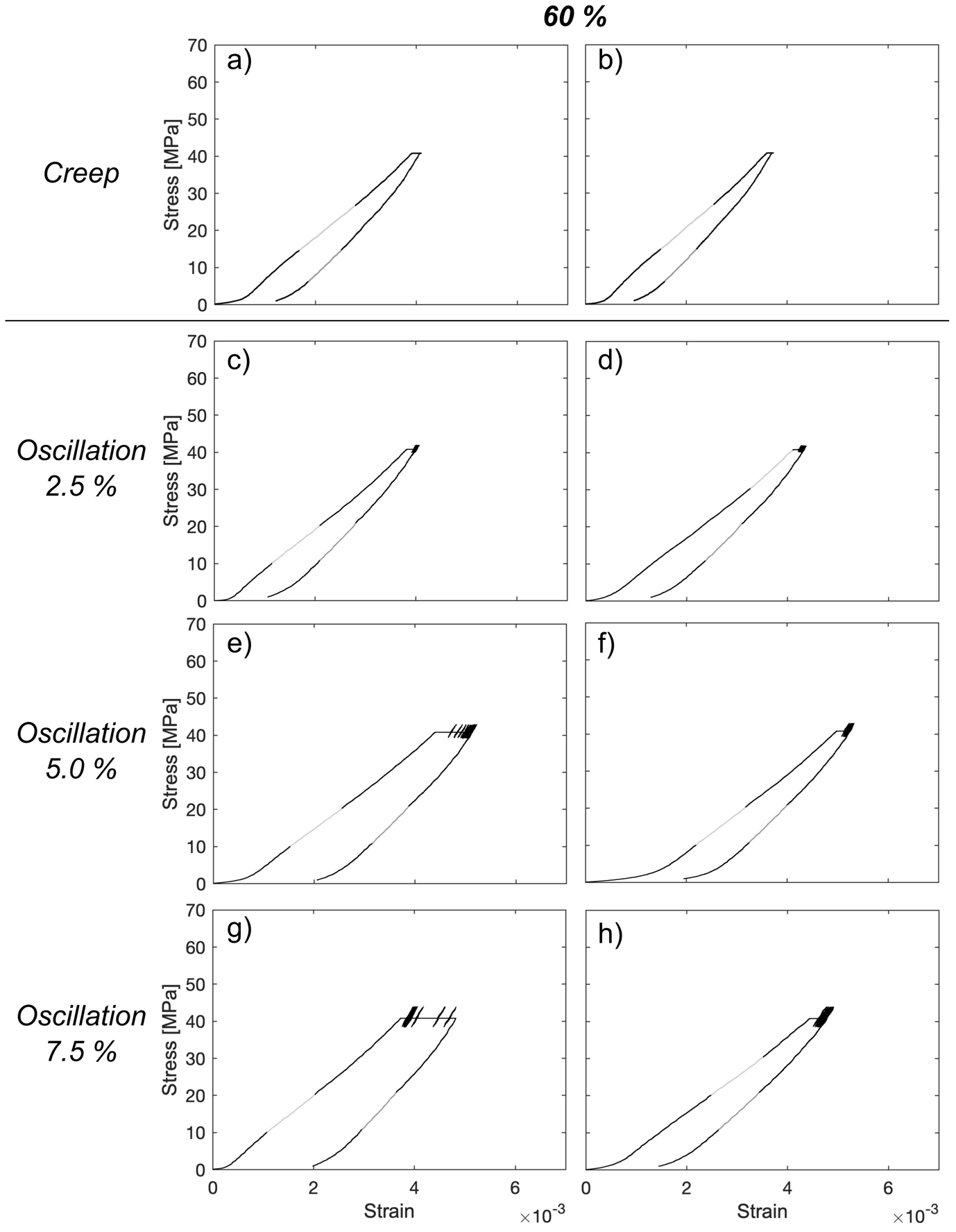


**Supplementary Figure 3. Stress-strain curves for samples tested at 60% of the expected UCS**. a), b) Data recorded during creep tests; c), d) Data recorded during oscillation tests with an amplitude of ±2.5%; e), f) Data recorded during oscillation tests with an amplitude of ±5%; g), h) Data recorded during oscillation tests with an amplitude of ±7.5%. The grey areas denote where the Young’s modulus was calculated during loading and unloading.


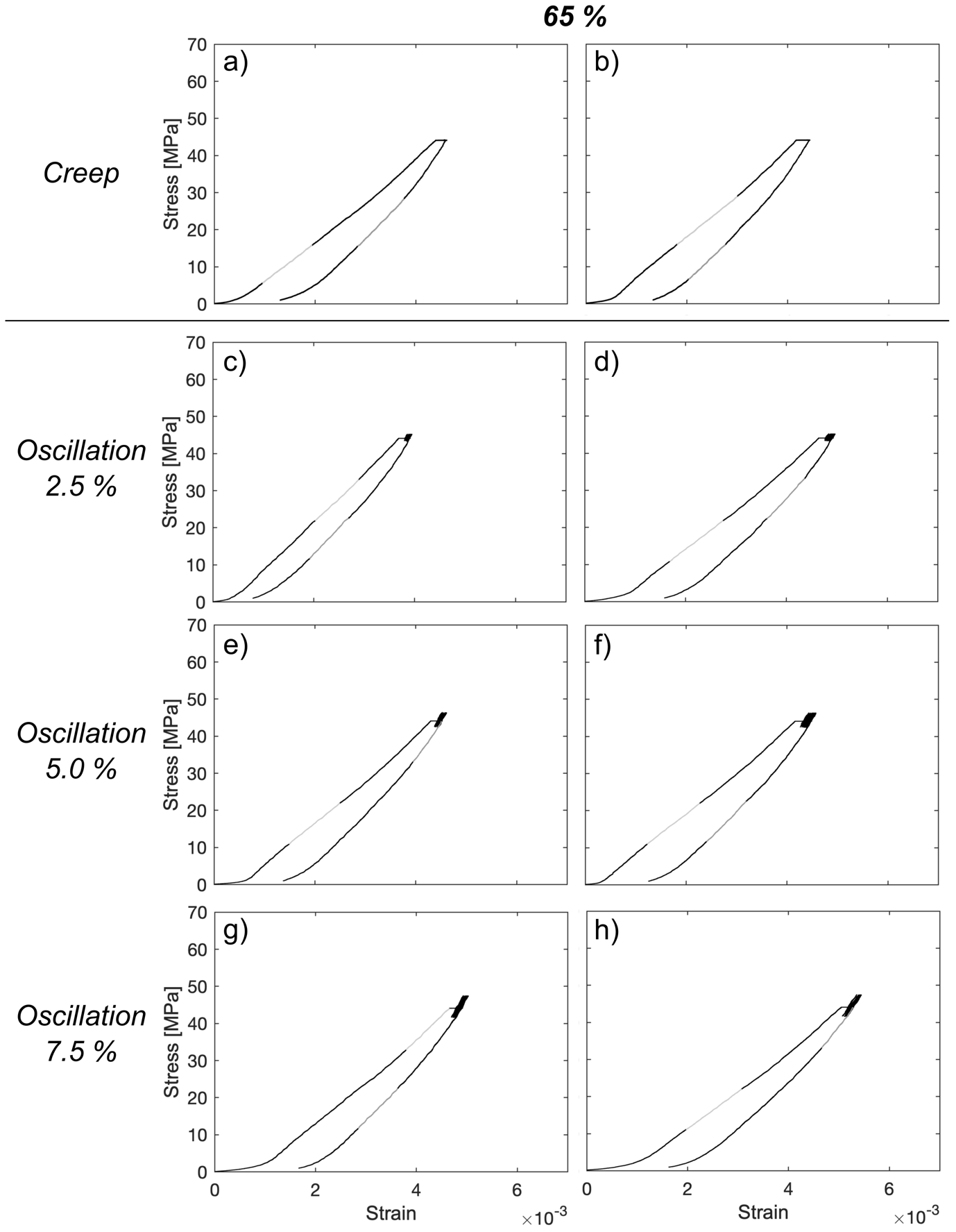


**Supplementary Figure 4. Stress-strain curves for samples tested at 65% of the expected UCS**. a), b) Data recorded during creep tests; c), d) Data recorded during oscillation tests with an amplitude of ±2.5%; e), f) Data recorded during oscillation tests with an amplitude of ±5%; g), h) Data recorded during oscillation tests with an amplitude of ±7.5%. The grey areas denote where the Young’s modulus was calculated during loading and unloading.


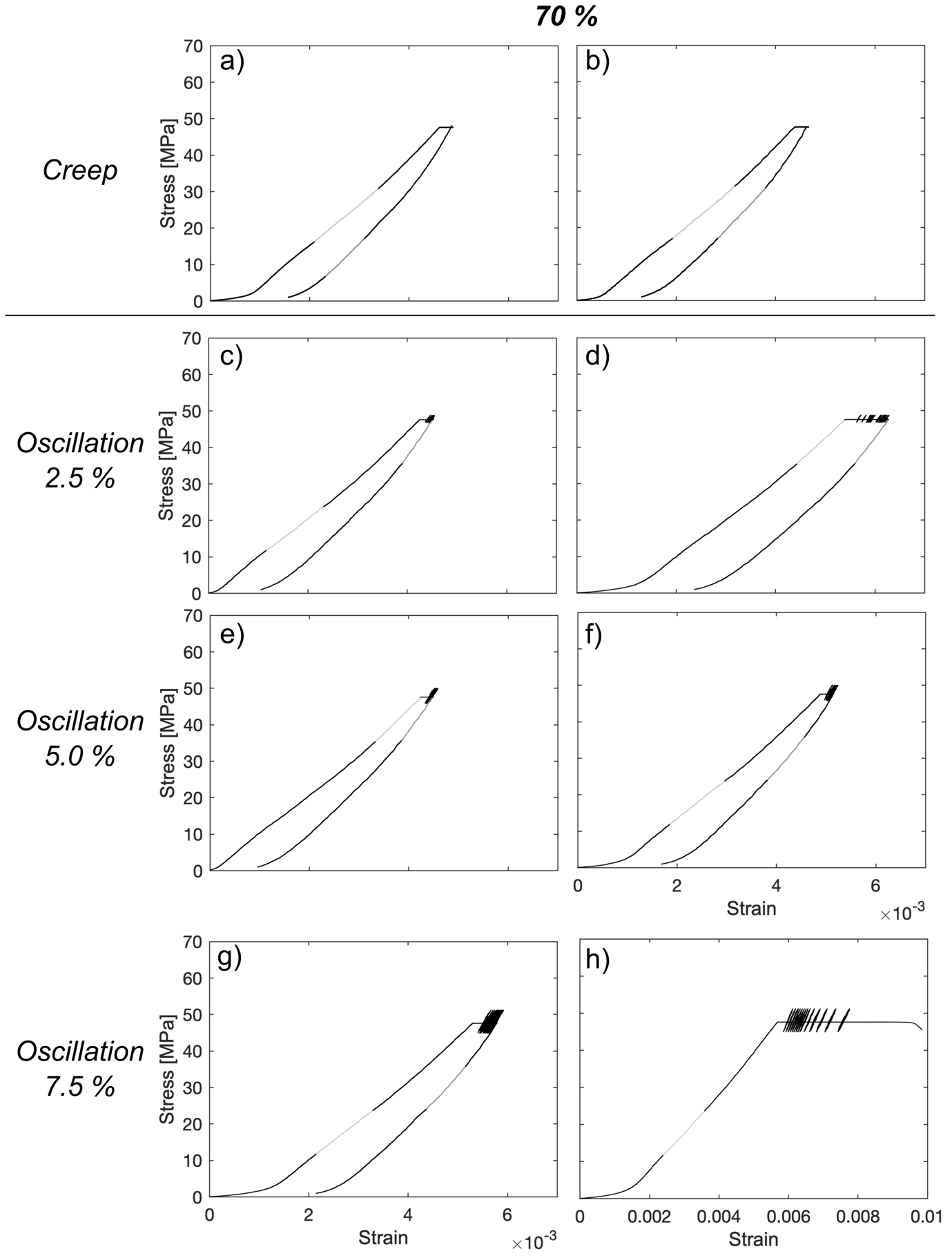


**Supplementary Figure 5. Stress-strain curves for samples tested at 70% of the expected UCS**. a), b) Data recorded during creep tests; c), d) Data recorded during oscillation tests with an amplitude of ±2.5%; e), f) Data recorded during oscillation tests with an amplitude of ±5%; g), h) Data recorded during oscillation tests with an amplitude of ±7.5%. The grey areas denote where the Young’s modulus was calculated during loading and unloading.

**Dynamic creep removal**

The effect of creep on strain is removed from oscillation experiments by:

1. Cutting the data to obtain the creep phase only and recording the first time and strain data point for all creep and oscillation tests signals.
2. Zeroing both time and strain by subtracting the first data point for all creep and oscillation tests signals.
3. Computing the average creep using the 2 samples at each load conditions (60, 65 and 70% of expected failure; Supplementary Fig. 8a), grey lines).
4. As each sample exhibits some variability, we then use least square regressions to model the average creep for a given time at each load condition (Supplementary Fig. 8a), blue lines).
5. Comparing the time values in the oscillation signals at a given load condition to the modelled signal and subtracting the corresponding modelled creep strain.
6. Adding the recorded time and strain first data point to “un-zero” the data (Supplementary Fig. 8b).


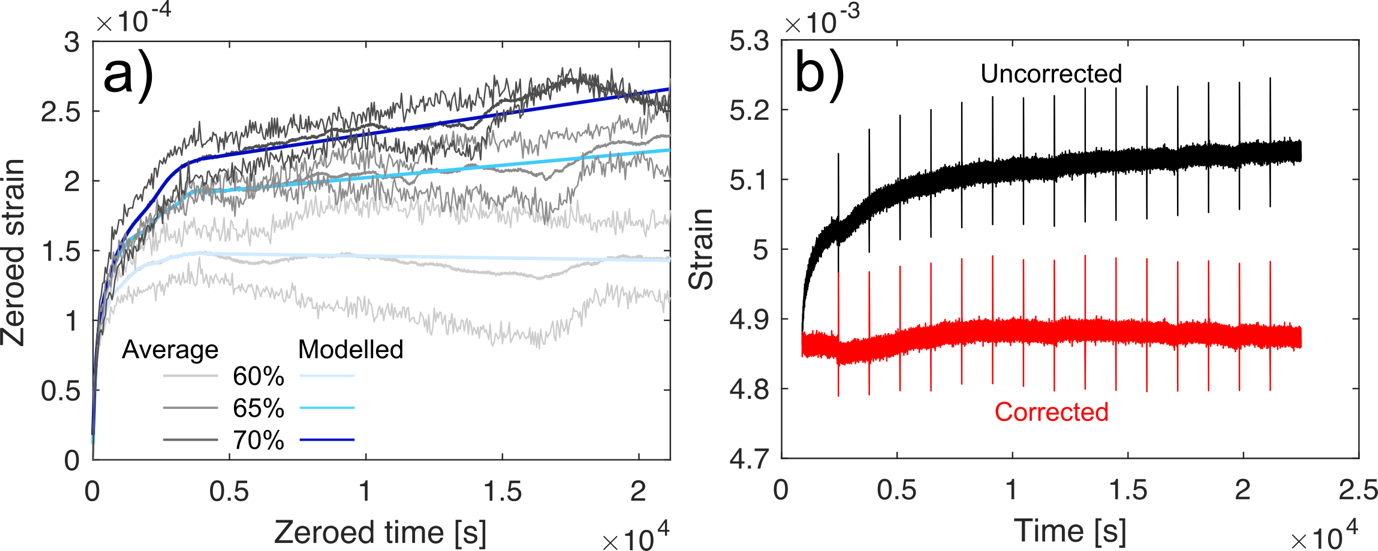


**Supplementary Figure 6. Dynamic creep removal**. a) Least square regressions calculated from average creep at each load condition; b) Effect of dynamic creep removal for a test at 65% of the UCS value and ±7.5% amplitude oscillations.


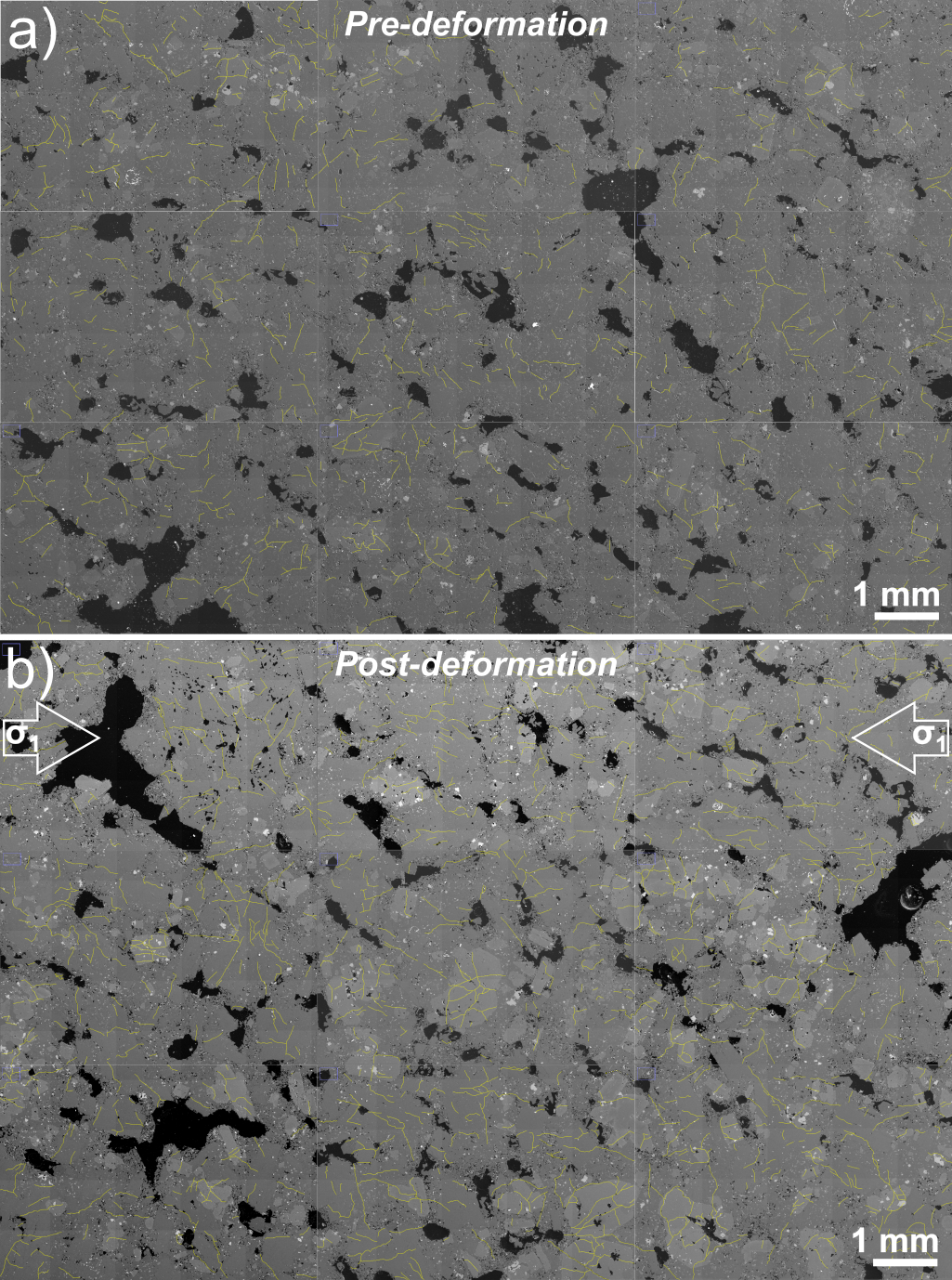


**Supplementary Figure 7. Fracture traces in intact and deformed samples**. a) Fracture traces in the undeformed material; b) Fracture traces in a sample deformed at a creep intensity of 70% of UCS and ±5% amplitude oscillations. These images provide the basis for the data presented in Figure 1f.

**
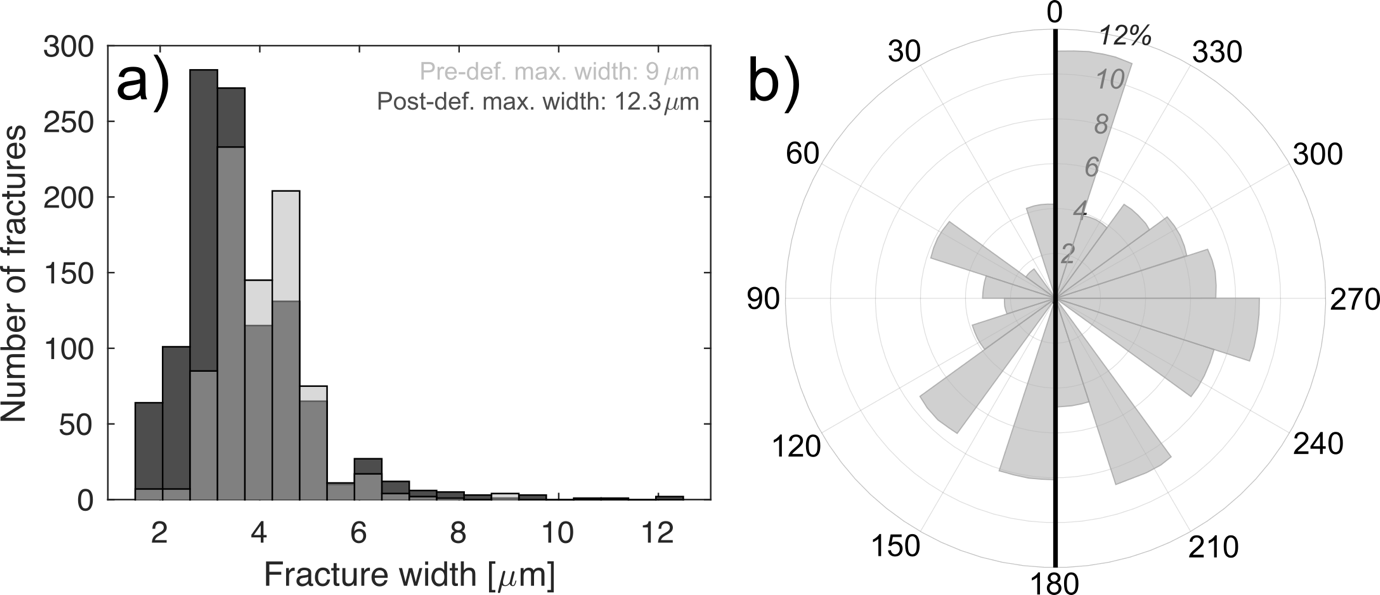
**

**Supplementary Figure 8. Fracture width and angles**. a) Fracture width in the undeformed material (light, transparent grey) and deformed material (dark grey; deformed at 70% of the UCS value; ±5% amplitude oscillations); b) Percent increase in each fracture angle after deformation. The black line denotes the axis of applied principal stress.
